# Distribution Patterns of Antibiotic Resistance Genes and Their Bacterial Hosts in a Manure Lagoon of a Large-Scale Swine Finishing Facility

**DOI:** 10.3390/microorganisms10112301

**Published:** 2022-11-20

**Authors:** Shahjahon Begmatov, Alexey V. Beletsky, Eugeny V. Gruzdev, Andrey V. Mardanov, Lubov B. Glukhova, Olga V. Karnachuk, Nikolai V. Ravin

**Affiliations:** 1 Institute of Bioengineering, Research Center of Biotechnology of the Russian Academy of Sciences, 119071 Moscow, Russia; 2 Laboratory of Biochemistry and Molecular Biology, Tomsk State University, 634050 Tomsk, Russia

**Keywords:** antibiotic resistance genes, metagenome, swine manure, microbiome, *Escherichia flexneri*, *Alcaligenaceae*

## Abstract

The spread of antibiotic resistance genes (ARGs) that are present in livestock manures, which are discharged into the environment, is a severe threat to human and animal health. Here, we used 16S rRNA gene profiling and metagenomic analysis to characterize microbial community composition and antibiotic resistance in a manure storage lagoon from a large-scale swine finishing facility. Manure samples were collected at intervals of two years. Both the prokaryotic community and the resistome were dominated by the *Firmicutes*, *Proteobacteria* and *Bacteroidota*. Metagenomic analysis of two samples revealed 726 and 641 ARGs classified into 59 and 46 AMR gene families. Besides multidrug efflux pumps, the predominating ARGs potentially encoded resistance to tetracyclines, macrolide–lincosamide–streptogramin, aminoglycosides, peptide antibiotics, rifamycin, chloramphenicol, and beta-lactams. Genes from all predominant AMR gene families were found in both samples indicating overall long-term stability of the resistome. Antibiotic efflux pumps were the primary type of ARGs in the *Proteobacteria*, while antibiotic target alteration or protection was the main mechanism of resistance in the *Firmicutes*, *Actinobacteriota* and *Bacteroidota*. Metagenome-assembled genomes (MAG) of four multidrug-resistant strains were assembled. The first MAG, assigned to *Escherichia flexneri*, contained 46 ARGs, including multidrug efflux pumps, modified porins, beta-lactamases, and genes conferring resistance to peptide antibiotics. The second MAG, assigned to the family *Alcaligenaceae*, contained 18 ARGs encoding resistance to macrolide–lincosamide–streptogramin, tetracyclines, aminoglycosides and diaminopyrimidins. Two other MAGs representing the genera *Atopostipes* and *Prevotella*, contained four and seven ARGs, respectively. All these MAGs represented minor community members and accounted for less than 0.3% of the whole metagenome. Overall, a few lineages originated from the gut but relatively rare in the manure storage lagoon, are the main source of ARGs and some of them carry multiple resistance determinants.

## 1. Introduction

Antibiotic-resistant bacteria (ARB) and antibiotic resistance genes (ARG) are a global threat to both humans and animals. Antimicrobials are traditionally used to prevent and treat bacterial infections in livestock. Antibiotics are not fully degraded by the animals, but rather excreted with urine and feces, which are often used as organic fertilizers on arable land. This agricultural practice causes the dissemination of ARB and ARG in the environment [1,2]. Swine manure is considered to be a “hotspot” of ARB [3,4], and its discharge has been referred to as the ARG pollution of soil [5]. Swine farms have been indicated as important sources of tetracycline and sulfonamide resistance reservoirs in the environment [6,7].

The best-studied bacteria in the resistome are enteric, which are associated with food- and water-borne diseases [8,9]. It is broadly agreed that multidrug-resistant *Enterobacteriaceae* can spread or disseminate ARGs from animal farms or clinics/hospitals and can adversely affect agriculture and public health [10]. While numerous studies have explored the ARG distribution in the model enteric bacterial pathogens [6,11], less attention has been paid to the resistome of free-living environmentally relevant bacteria receiving ARG from gut ARB. Manure storage lagoons used for the collection and primary treatment of swine manure is a system in which gut microbes may transmit ARG to environmentally relevant bacteria. Non-pathogenic environmental microorganisms have been suggested as a massive reservoir of antibiotic resistance genes [12]. Previously, we demonstrated the occurrence of ARG in *Desulfovibrio desulfuricans* L4, a sulfate-reducing bacterium, isolated from waste of a large-scale swine finishing facility [13]. Strain L4 contained a plasmid with a multidrug-resistance gene cassette, containing tetracycline-, streptomycin-, and sulfonamide-resistance genes. The horizontal transfer of this plasmid was suggested from *Enterobacteriaceae*, namely *Shigella flexneri*, which harbored a nearly identical plasmid.

The aim of the present study was to perform a metagenomics analysis of manure slurry from the same large-scale finishing facility to profile antibiotic resistome and predict ARG dissemination in the environmentally relevant prokaryotes. Here, we report microbial community composition and resistomes of manure samples collected in different years.

## 2. Materials and Methods

### 2.1. Study Site, Sampling, and Physicochemical Parameters Measurements

Swine manure was collected from a large-scale swine finishing facility, ‘Tomskii’, with a capacity of 176,000 hogs a year and located in the close vicinity of Tomsk, the capital city of the Tomsk region in Western Siberia, Russia. Manure was sampled on 2 November 2021 from the manure storage lagoon receiving cumulative slurry from the facility (the Tom2 sample). The second sample, the near-bottom manure sediment slurry, was collected from the same storage lagoon (the Tom3 sample) on 18 October 2019 and used for *Desulfovibrio* isolation [13]. Total genomic DNA from manure slurry samples was extracted using a Power Soil DNA isolation kit (Qiagen, Hilden, Germany) and stored at −20 °C.

### 2.2. Sequencing of the 16S rRNA Gene Fragments and Bioinformatics Analysis of Microbial Community Composition

PCR amplification of 16S rRNA gene fragments comprising the V3–V4 variable regions was carried out using the universal prokaryotic primers PRK 341F (5′-CCTAYG GGDBGCWSCAG) and PRK 806R (5′-GGA CTA CNVGGG THTCTAAT) [14]. The PCR fragments were sequenced on Illumina MiSeq in a paired reads format (2 × 300 nt). Pairwise overlapping reads were merged using FLASH v.1.2.11 [15]. The 16S rRNA gene sequences were clustered into operational taxonomic units (OTUs) at 97% identity using the USEARCH v. 11 program [16]. Low-quality reads, chimeric sequences, and singletons were removed by the USEARCH algorithm. A total of 24,237 (Tom2) and 26,062 (Tom3) sequences passed all filters. To calculate OTU abundances, all obtained reads were mapped to OTU sequences at a 97% global identity threshold by USEARCH. The taxonomic assignment of OTUs was performed by searching against the SILVA v.138 rRNA sequence database using the VSEARCH v. 2.14.1 algorithm [17].

### 2.3. Metagenomic Sequencing and Identification of ARG

Total DNA isolated form manure slurry sample was sequenced using the Illumina HiSeq2500 platform according to the manufacturer’s instructions (Illumina Inc., San Diego, CA, USA). The sequencing of a paired-end (2 × 150 bp) TruSeq DNA library generated 192,103,014 read pairs (~57.6 Gb) for the Tom2 sample and 108,273,070 read pairs (~32.5 Gb) for the Tom3 sample. Adapter removal and trimming of low-quality sequences (Q < 30) were performed using Cutadapt v.3.4 [18] and Sickle v.1.33 (https://github.com/najoshi/sickle, accessed on 14 October 2022), respectively. The reads were de novo assembled into contigs using the MEGAHIT v.1.2.9 program [19].

The obtained contigs were binned into metagenome-assembled genomes (MAGs) using MetaBAT v. 2.15 (Kang et al., 2019). The assembled MAGs were taxonomically classified using the Genome Taxonomy Database Toolkit (GTDB-Tk) v.1.5.0 [20] and Genome Taxonomy database (GTDB) [21]. CheckM 1.1.3 [22] was used to check completeness and contamination values of obtained MAGs. The open reading frames (ORFs) in contigs were predicted using Prodigal v2.6.3 (Hyatt et al., 2010). To identify putative ARGs, we aligned the predicted protein sequences of ORFs against the Comprehensive Antibiotic Resistance Database (CARD, https://card.mcmaster.ca/, accessed on 14 October 2022) [23,24,25] using the BLASTP tool with e-value < 1 × 10^−5^ [23]. An ORF was annotated as an ARG if the best BLASTP hit showed at least 80% identity over a query coverage of 85% [23]. The identified ARG-like ORFs were then assigned to various AMR gene families, drug classes and resistance mechanisms categories according to the CARD. The taxonomy of the ARG-carrying contigs was assigned using the Kaiju webserver with the NCBI NR protein database as a reference [26].

## 3. Results

### 3.1. Composition of Tom2 Manure Slurry Microbiome Revealed by 16S rRNA Gene Profiling

**Figure 1:**
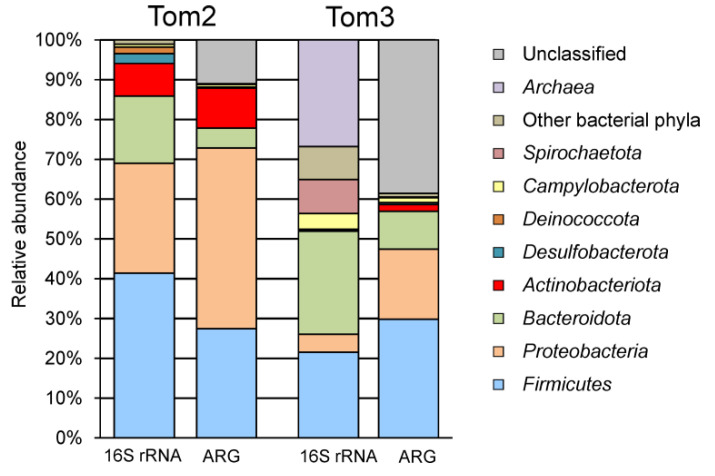
Compositions of microbial communities (16S rRNA) and resistomes (ARG) in the manure storage lagoon at the domain/phylum level. Taxonomic composition of the resistome is shown according to predicted taxonomy of the ARG-carrying contigs.

To characterize the taxonomic compositions of microbial communities a total of 37,335 sequences of 16S rRNA gene fragments were determined and clustered into 593 OTUs at the level of 97% sequence identity. Taxonomic assignment of OTUs revealed the presence of 17 phylum-level lineages of Bacteria and Archaea, recognized in the Genome Taxonomy Database (GTDB). Archaea were represented by a single OTU assigned to Candidatus *Methanoplasma*, which accounted for only 0.01% of all 16S rRNA gene sequences. Bacterial community was dominated by the *Firmicutes* (41.4% of 16S rRNA gene reads), other major groups were the *Proteobacteria* (27.6%), *Bacteroidota* (16.9%), *Actinobacteriota* (8.2%), *Desulfobacterota* (2.5%), *Deinococcota* (1.6%) and *Campylobacterota* (0.8%) (Figure 1). Other phyla altogether accounted for only 1% of the community.

Most of *Firmicutes* were assigned to the class *Bacilli* (37.0%), which was dominated by unclassified lineages of the family *Bacillaceae* (about 28%) phylogenetically distant from cultured species, and the genus *Atopostipes* (*Carnobacteriaceae*, *Lactobacillales*) accounting for about 7.5%. *Atopostipes* sp. are typical inhabitants of various composts [27,28]. Classes *Clostridia* and *Limnochordia* were less numerous (2.2% and 2.1%, respectively) and most of the corresponding OTUs were not classified even at the family level representing phylogenetically distant uncultured groups.

*Proteobacteria* mostly belonged to the class gamma (27.3%) and comprised 157 OTUs. However, the majority of proteobacterial 16S rRNA gene sequences belonged to only two OTUs. One of them, comprising 16.1% of all 16S rRNA gene reads, was assigned to the family *Alcanivoracaceae* (*Pseudomonadales*), while the second one (5.1%) belonged to the *Alcaligenaceae* (*Burkholderiales*); both OTUs were not classified at the genus level.

The phylum *Bacteriodetes* was mostly represented by the families *Flavobacteriaceae* (6.6%), *Sphingobacteriaceae* (5.5%) and *Marinilabiliaceae* (3.4%). Most abundant OTUs belonged to uncultured candidate genera. The phylum *Actinobacteriota* mostly comprised the members of *Corynebacteriales* and *Micrococcales*, and nearly a half of *Actinobacteriota* belonged to the genus *Corynebacterium* (4.6%), which was reported in swine manure compost [29]. *Desulfobacterota* was represented by four OTUs phylogenetically distant from cultured lineages of this phylum. As revealed by GenBank searches, the most abundant OTU (2.3%) showed only 82.5% sequence identity to the closest cultured relative, *Natronanaerobius thermophilum*, and 96.6% identity to environmental clone OTU_112 (GenBank MH312167) found in river sediments. Conversely, all *Deinococcota* OTUs belonged to the known genus *Truepera*, frequently found in the bacterial communities during swine manure composting [30]. Finally, the phylum *Campylobacterota* mostly comprised known genera of sulfur-oxidizing bacteria, *Sulfurimonas* and *Sulfurospirillum*. Their growth was probably supported by hydrogen sulfide generated by sulfate reducing bacteria [13].

### 3.2. Composition of Tom3 Manure Slurry Microbiome

The structure of the microbial community of the near-bottom sediment slurry, the Tom3 sample, was briefly reported in [13] and will be presented in more detail below. Unlike Tom2, in the Tom3 sample, Archaea accounted for about 27% of all 16S rRNA gene sequences and were represented mostly by methanogens of the orders *Methanobacteriales*, *Methanomicrobiales* and *Methanomassiliicoccales*. The high relative abundance of methanogens is probably related to anaerobic conditions in the sediments. Bacterial community was dominated by the *Bacteroidota* (25.9%) and the *Firmicutes* (21.5%), and other major groups were the *Spirochaetota* (8.6%), *Proteobacteria* (4.5%) and *Campylobacterota* (3.9%), while *Actinobacteriota and Desulfobacterota* were detected in minor amounts (Figure 1).

The phylum *Bacteriodetes* was mostly represented by taxa typical of animal digestive tracts, assigned to the families *Bacteroidaceae, Rikenellaceae, Prevotellaceae* and *Dysgonomonadaceae*. Most of *Firmicutes* were assigned to the class *Clostridia* (13.5%) which was dominated by the families *Ruminococcaceae* and *Lachnospiraceae*, and various uncultured lineages. Classes *Bacilli* and *Negativicutes* were less numerous (7.5% and 0.5%, respectively). *Proteobacteria* mostly belonged to the class gamma (4.3%) of the orders *Enterobacterales* (2.4%) and *Pseudomonadales* (1.4%). Interestingly, two proteobacterial OTUs highly abundant in the Tom2 sample were not found in Tom3. *Spirochaetota* were mostly represented by the genus *Sphaerochaeta* (5.4%) and *Treponema* (3.0%), typically found in pig gut microbiomes [31]. Finally, the phylum *Campylobacterota* mostly comprised the family *Arcobacteraceae* (3.5%), while sulfur-oxidizing bacteria of the genera *Sulfurimonas* and *Sulfurospirillum* were less numerous.

### 3.3. Characterization of the Tom2 Manure Resistome

Metagenome sequencing of the Tom2 manure sample resulted in assembly of 1,424,703 contigs with N50 size of 1452 bp and a total length of 1359 Mb, the maximal contig length was 749,306 bp. In total, 726 ARGs classified into 59 AMR gene families recognized in CARD were identified in 673 contigs (Supplementary Table S1). The most numerous AMR gene families were resistance-nodulation-division (RND) antibiotic efflux pumps (141 genes), major facilitator superfamily (MFS) antibiotic efflux pumps (96 genes), Erm 23S ribosomal RNA methyltransferase (70 genes), tetracycline-resistant ribosomal protection protein (54 genes), Pmr phosphoethanolamine transferase (42 genes), vga-type ABC-F protein (41 genes) and rifamycin-resistant beta-subunit of RNA polymerase (33 genes). The detected resistance genes represented all major resistance mechanisms: antibiotic inactivation (147 genes), antibiotic efflux or reduced permeability to antibiotic (267 genes), and antibiotic target alteration, replacement or protection (312 genes).

**Figure 2:**
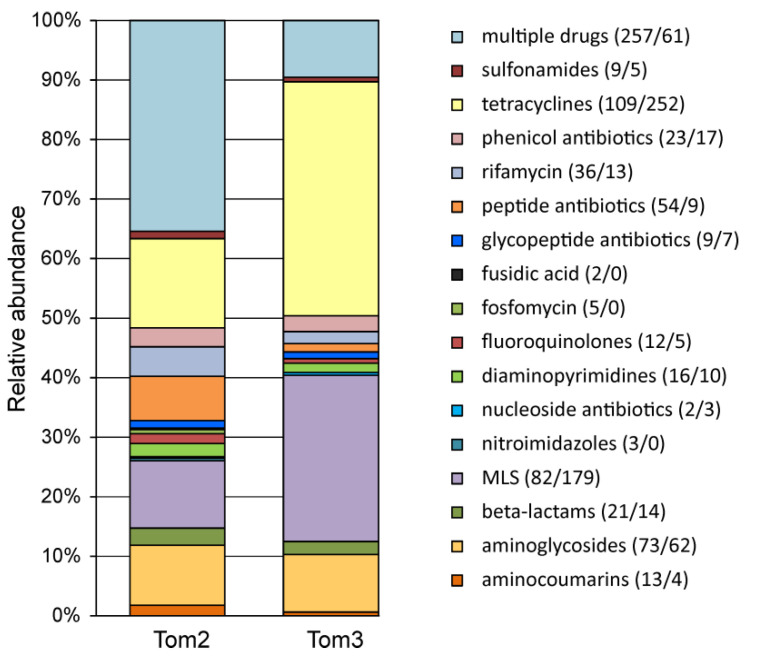
Relative abundances of ARG identified in manure samples Tom2 and Tom3 categorized by class of resistance. Numbers of genes are shown in parentheses (Tom2/Tom3).

Tetracyclines are the most commonly used antibiotics for animal growth promotion and disease control [7,32,33,34] and ARG profiles obtained from our sample indicated that resistance to this antibiotic was most frequent among the detected ARGs (109 genes) (Figure 2). Other frequently found ARG drug classes included MLS antibiotics (macrolide–lincosamide–streptogramin, 82 genes), aminoglycosides (73 genes), peptide antibiotic (54 genes), rifamycin (36 genes), phenicol antibiotics (e.g., chloramphenicol, 23 genes), and beta-lactams (21 genes). Nevertheless, many ARGs encoded multidrug efflux pumps and were predicted to confer resistance to several classes of antibiotics.

Taxonomic assignment of contigs carrying ARGs was estimated by Kaiju. Most ARG were assigned to the *Proteobacteria* (330 genes, 45% of the total) followed by *Firmicutes* (199, 27%), *Actinobacteriota* (73, 10%), *Bacteroidota* (36, 5%), *Campylobacterota* (5, 0.7%), *Desulfobacterota* (2, 0.3%) and *Acidobacteriota* (1, 0.1%); taxonomic affiliation of other 80 ARG remained uncertain (Figure 1).

**Figure 3:**
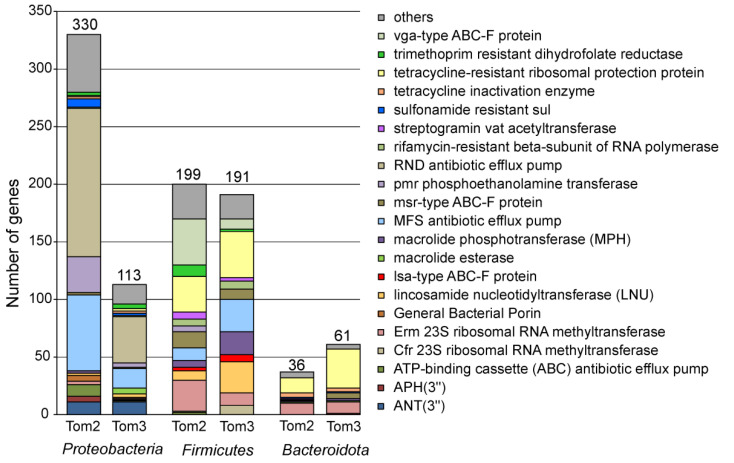
Abundances of ARG identified in the Tom2 and Tom3 manure samples categorized by AMR gene family. Numbers of ARG assigned to particular family are shown for the *Proteobacteria*, *Firmicutes* and *Bacteroidota*. Others, ARG families comprising less than 5 ARG in these phyla.

The majority of ARG assigned to the *Proteobacteria* represented antibiotic efflux pumps of the RND (129 genes), MFS (66 genes) and ATP-binding cassette (ABC) (10 genes) families (Figure 3, full data are shown in Supplementary Table S1).

They were predicted to confer resistance to various compounds, including fluoroquinolones, tetracyclines, macrolides, diaminopyrimidine antibiotics, phenicol antibiotics, beta-lactams, aminoglycosides, aminocoumarin antibiotics, disinfecting agents and antiseptics. Resistance to aminoglycosides based on antibiotic inactivation could be conferred by ARGs of AAC(3), AAC(6′), APH(3′), ANT(3″), ANT(4′), APH(4) and APH(6) families (30 genes in total). A total of 31 ARG of the Pmr phosphoethanolamine transferase family (mostly *ugd* genes) were predicted to determine resistance to peptide antibiotics such as polymyxin. Resistance to beta lactams such as carbapenem, cephalosporin and penam is probably determined by various beta-lactamases (15 genes of *ampC, CARB, CMY, CTX-M, EC, OXA* and *SRT* types), while seven sul genes (*sul1*, *sul2*, and *sul3*) could enable resistance to sulfonamides. Although MFS family efflux pump were most numerous among tetracycline resistance determinants, two *tet(X6)* genes determined antibiotic inactivation and *tet*(57) gene coding for tetracycline-resistant ribosomal protection protein were found as well. Considering other drugs, specific resistance genes were found for lincosamides (lincosamide nucleotidyltransferase, two genes), macrolides (macrolide phosphotransferase, 2 genes), chloramphenicol (chloramphenicol acetyltransferase, four genes), fluoroquinolones (quinolone resistance protein Qnr, two genes), diaminopyrimidine antibiotics (trimethoprim resistant dihydrofolate reductase Dfr, three genes), and streptothricin (streptothricin acetyltransferase).

More than a quarter of detected ARGs were assigned to *Firmicutes* (Figure 3). Contrary to the *Proteobacteria*, antibiotic target alteration/protection was the most frequent resistance mechanism (146 genes), while antibiotic inactivation and efflux were less common (39 and 14 genes, respectively). The most numerous were genes of the ABC-F protein families, conferring resistance through ribosomal protection in Gram-positive bacteria [35]. A total of 40 genes of the vga-type ABC-F proteins and three genes encoding the lsa-type ABC-F proteins are known to provide resistance to lincosamides and streptogramin A antibiotics. Fourteen genes of the msr-type ABC-F protein family are responsible for resistance to macrolides and group B streptogramins, and one *optrA* gene confers resistance to oxazolidinones and phenicols [35]. The lsa-type and vga-type ABC-F proteins were found only in the *Firmicutes*. Resistance to tetracyclines could be enabled by 31 genes of tetracycline-resistant ribosomal protection protein family (*tetT*, *tetS*, *tetO*, *tetM* and *tet32*) and three genes encoding MFS family efflux pumps (*tet(L)* and *tet(40)*). The presence of 27 genes encoding Erm 23S ribosomal RNA methyltransferase, three genes for non-erm 23S ribosomal RNA methyltransferase (G748) and one gene for Cfr 23S ribosomal RNA methyltransferase indicates resistance to several antibiotics, such as lincosamides, macrolides, and streptogramins through target alteration. Resistance to aminoglycosides based on antibiotic inactivation could be conferred by ARGs of AAC(6′), APH(2″), ANT(4′), ANT(6), ANT(9), APH(2″), APH(3′) families (11 genes in total). There are 10 genes for trimethoprim resistant dihydrofolate reductase Dfr, eight genes for lincosamide nucleotidyl transferase (LNU), five *ugd* genes for phosphoethanolamine transferase (Pmr), four genes for chloramphenicol acetyltransferase, six genes for macrolide phosphotransferase, six genes for streptogramin Vat acetyltransferase, and two genes of the glycopeptide resistance gene cluster (vanR). Resistance to rifamycin could be conferred by rifamycin-resistant beta-subunit of RNA polymerase (6 *rpoB* genes) and rifampin phosphotransferase (two *rphB* genes). The presence of two genes for FusB-type target protecting proteins, fosfomycin thiol transferase gene *fosD*, and *blaZ* beta-lactamase gene indicates resistance to fusidic acid, fosfomycin and penam. Genes encoding antibiotic efflux pumps were less numerous among ARGs of the *Firmicutes*. Addition to aforementioned three tetracycline efflux pumps, there are 10 other genes encoding the MFS superfamily transporters predicted to confer resistance to macrolides, fosfomycin, chloramphenicol, and various disinfecting agents and antiseptics. A single *bcrA* gene encoding an ABC transporter probably confers bacitracin resistance.

Among 73 actinobacterial ARGs, the most numerous was rifamycin-resistant beta-subunit of RNA polymerase *rpoB* (24 genes), followed by MFS tetracycline efflux pumps (*tet(33), tet(Z)* and *tetB(48)*, a total of 14 genes), and Erm 23S ribosomal RNA methyltransferase conferring resistance to lincosamides and macrolides (11 genes) (Figure 3). RbpA gene encoding RNA polymerase-binding protein also provides resistance to rifamycin. Resistance to macrolides could be determined by MtrA efflux pump of the RND family (10 genes) and macrolide phosphotransferase MphO (three genes). Six *vanR* glycopeptide resistance gene of the Van cluster probably confer resistance to drugs of this class. The presence of two *cmx* genes for MFS superfamily exporter indicates resistance to chloramphenicol.

Interestingly, all 36 ARGs assigned to the members of the *Bacteroidota* confer resistance via antibiotic inactivation or target alteration/protection, while drug efflux pumps were not found (Figure 3). Resistance to tetracyclines was the most common, it was determined by tetracycline-resistant ribosomal protection proteins (two *tetQ* and 11 *tet36* genes) or through tetracycline inactivation (*tet(X4), tet(X5), tet(X6)*, and *tetX*). Ten *erm(35)* genes encoding the Erm 23S ribosomal RNA methyltransferase likely determine resistance to lincosamides and macrolides. Four *aadS* genes (ANT(6) family) could be responsible for resistance to aminoglycoside antibiotics. Other resistance determinants were MphG macrolide phosphotransferase, EreD macrolide esterase, msr-type ABC-F protein (confers resistance to macrolides and streptogramin), streptogramin Vat acetyltransferase and sulfonamide resistance protein Sul2.

### 3.4. Diversity of ARG from the Tom3 Manure Sample

Metagenomic analysis of the Tom3 manure sample revealed 641 ARG classified into 46 AMR gene families (Supplementary Table S2). Resistance to tetracyclines was most frequent in the Tom3 resistome (252 genes) followed by MLS antibiotics (179 genes) and aminoglycosides (62 genes) (Figure 2). Interestingly, ARGs encoding multidrug efflux pumps were less frequent than in the Tom2 sample (61 genes), as well as ARGs conferring resistance to aminocoumarins, fluoroquinolones, peptide antibiotics and rifamycin (Figure 2).

Taxonomic assignment of contigs carrying ARGs by Kaiju enabled to classify 394 of 641 detected ARG (Figure 1). Most of them were assigned to the *Firmicutes* (191, 30% of the total), *Proteobacteria* (113 genes, 18%) and *Bacteroidota* (61 genes, 10%), while none represented archaea. For the *Firmicutes*, the most frequent resistance mechanism was antibiotic target alteration/protection (98 genes), followed by antibiotic inactivation (63 genes) and efflux (30 genes). ARGs enabling resistance to MLS antibiotics were most numerous and included lincosamide nucleotidyltransferase LNU (27 genes), Erm and Cfr 23S ribosomal RNA methyltransferases (11 and 8 genes, respectively), vga-type (nine genes), lsa-type (six genes) and msr-type (nine genes) ABC-F proteins, macrolide phosphotransferase MPH (20 genes) and streptogramin Vat acetyltransferase. Resistance to tetracyclines could be conferred by tetracycline-resistant ribosomal protection proteins (40 genes, mostly *tet(T)*) and MFS superfamily efflux pumps (24 genes). ARGs of AAC(6′), APH(2″), ANT(4′), ANT(6), and ANT(9) families (10 in total) could be responsible for inactivation of aminoglycoside antibiotics. The presence of seven *rpoB* genes for the rifamycin-resistant beta-subunit of RNA polymerase indicates resistance to this drug. In addition, there are three genes for chloramphenicol acetyltransferase (CAT), four genes of glycopeptide resistance *van* cluster, two genes for trimethoprim resistant dihydrofolate reductase Dfr, two genes for streptothricin acetyltransferase (SAT), two *optrA* genes encoding ABC-F subfamily ATP-binding cassette ribosomal protection proteins, and six efflux pumps.

Like in the metagenome of Tom2 sample, the majority of ARG assigned to the *Proteobacteria* represented antibiotic efflux pumps (61 genes) predicted to confer resistance to various drugs. Seventeen genes of the AAC(3), AAC(6′), ANT(3″), APH(3′) and APH(4) families could enable inactivation of aminoglycosides. Resistance to MLS antibiotics could be determined by lincosamide nucleotidyltransferase (three genes), Erm and Cfr 23S ribosomal RNA methyltransferases (two genes), macrolide esterase (five genes) and phosphotransferase (one gene). Specific resistance genes were also found for diaminopyrimidines, fluoroquinolones, beta lactams, peptide antibiotics, chloramphenicol, sulfonamide, rifamycin and tetracycline, like in the Tom2 manure sample.

The inventory of ARGs among the *Bacteroidetes* was similar to that observed in the Tom2 sample. The most common was resistance to tetracyclines determined by tetracycline-resistant ribosomal protection proteins (34 genes, mostly *tet(Q)* and *tet(36)*) or tetracycline inactivation enzyme (three genes). Genes encoding the Erm 23S ribosomal RNA methyltransferase (10 genes), msr-type ABC-F protein (five genes), macrolide phosphotransferase (two genes) and macrolide esterase (one gene) likely determine resistance to MLS antibiotics. Other resistance determinants were aminoglycoside phosphotransferases (APH(6) and APH(3″)), CfxA beta-lactamase (three genes) and sulfonamide resistance protein Sul2.

### 3.5. Multidrug Resistant Strains

To obtain MAGs of microbial community members, we sequenced the metagenomes of manure samples using Illumina technique and binned obtained contigs into MAGs. Since we were interested in genomes containing genetic determinants of multiple drug resistance, only MAGs with more than 50% completeness and less than 25% contamination were selected for further analysis. Of the 140 such MAGs obtained for the Tom2 sample, 24 contained at least one ARG which can serve as a rough estimate of the proportion of resistant strains in the community. Twenty MAGs contained one gene each, two MAGs contained two ARGs and two genomes contained 18 and 46 resistance genes, apparently representing multidrug-resistant strains (Table 1).

The first genome, MAG-8, with an estimated 63% completeness and 2% possible contamination harbored 46 ARGs. This MAG accounted for only 0.03% of the whole metagenome. Analysis of the taxonomic affiliation of this MAG showed that it belongs to the genus *Escherichia* and showed 98.50% ANI with *Escherichia* (*Shigella*) *flexneri* strain 2a (GCA_002950215.1) therefore representing this species. Most of resistance genes encode antibiotic efflux pumps of RND (22 genes), MFS (12 genes) and ABC (three genes) families, conferring resistance to various drugs including aminocoumarins, aminoglycosides, fluoroquinolones, and macrolides. Three genes encode porins with reduced permeability to beta-lactams. In addition, there are *pmrF* and *eptA* genes (phosphoethanolamine transferase), and *bacA* gene (undecaprenyl pyrophosphate related protein) conferring resistance to peptide antibiotics, and three beta-lactamase genes (*EC-14, EC-5* and *ampC*) predicted to confer resistance to cephalosporins.

MAG-42, carrying 18 ARGs, was estimated to be 81% complete, with 25% possible contamination. The share of this MAG in the whole metagenome was estimated in 0.3%. Although such a high contamination did not allow its taxonomic identification using GTDB tools, 123 of 133 conservative marker genes showed best BLASTP hits with proteins form the members of the family *Alcaligenaceae* (*Proteobacteria*, *Burkholderiales*). Although *Alcaligenaceae* were among the dominant groups according to 16S rRNA gene profiling data (see above), this MAG represented a low abundance phylotype of this family. Eight ARGs were predicted to determine resistance to macrolide–lincosamide–streptogramin antibiotics, namely lincosamide nucleotidyltransferase (*lnuA*) enabling inactivation of lincosamide, Erm 23S (five genes) and non-erm (two genes) ribosomal RNA methyltransferases protecting their target. Resistance to tetracyclines could be provided by tetracycline-resistant ribosomal protection proteins (three genes) and an MFS efflux pump (*tet(L)*). Two aminoglycoside nucleotidyltransferases (ANT(6) and ANT(9)) and two aminoglycoside phosphotransferases (APH(2″) and APH(3′)) probably determine resistance to aminoglycoside antibiotics. In addition, this MAG contains two trimethoprim resistant dihydrofolate reductase genes (*dfrG*) responsible for resistance to diaminopyrimidins.

Similarly, in the case of the Tom3 sample, of the 172 MAGs with more than 50% completeness and less than 25% of contamination, 22 contained at least one ARG: 18 MAGs contained single ARG, two MAGs contained two ARGs, and two MAGs representing multidrug-resistant strains harbored four and seven ARGs. MAG Tom3-16, carrying four ARGs and obtained with 88% completeness and 5% contamination, was assigned to the genus *Atopostipes* (*Firmicutes, Bacilli*). This genome contains genes for tetracycline-resistant ribosomal protection protein (*tet(M)*), lincosamide nucleotidyltransferase LNU, chloramphenicol acetyltransferase CAT and kanamycin nucleotidyltransferase (*ANT(4′)-Ib*). The second MAG Tom3-259, assigned to the genus *Prevotella* (*Bacteroidota*), was estimated to be 90% complete, with 12% possible contamination. This MAG contained two genes for lincosamide nucleotidyltransferase LNU, two genes for tetracycline-resistant ribosomal protection protein, macrolide phosphotransferase gene (*mphB*), an aminoglycoside nucleotidyltransferase gene (*ANT(6)-Ia*) and *tetA(P)* gene of tetracycline efflux pump. Each of these MAGs accounted for less than 0.2% of the whole metagenome and therefore represented a low abundance phylotypes.

## 4. Discussion

In this work, we characterized the composition of the microbial community and the resistome of two swine manure samples collected from a storage lagoon. In the fecal swine matter *Firmicutes* is known to be the dominating phylum followed by the *Bacteroidota* and *Actinobacteriota* [35,36,37]. Likewise, *Firmicutes* and *Bacteroidota* predominated in the analyzed storage lagoon, but the second most numerous bacterial phylum in the Tom2 sample was the *Proteobacteria* (27.6%). Since the storage lagoon contains manure at different stages of degradation, occurring both under aerobic and anaerobic conditions, it can be assumed that particular lineages of *Proteobacteria* predominantly multiply in the lagoon, and only a minor part of *Proteobacteria* represented the microbiome of the swine gut. An increase in the relative abundance of the *Proteobacteria* during composting of swine manure has been reported in other studies [33,37,38]. Interestingly, in the Tom2 sample, most of the proteobacterial 16S rRNA gene sequences (21.2%) belonged to only two OTUs. The most numerous OTU was only distantly related to both cultured and uncultured sequences (the best GenBank hit was from *Alcanivorax limicola* JB21 with 94.2% identity). The corresponding MAG was assigned to the family *Alcanivoracaceae*, but was not classified at the genus level. Members of this family are known to be involved in degradation of alkanes (e.g., [39]), and are not typical members of gut microbiomes. This phylotype probably originated from the environment and proliferated already in the storage lagoon. These two phylotypes were absent in the Tom3 sample and, accordingly, the share of *Proteobacteria* in this sample was significantly lower (4.5% vs. 27.6%, Figure 1). Nevertheless, most other proteobacterial lineages identified in the Tom2 and Tom3 samples (e.g., *Burkholderiales, Pseudomonadales*, *Enterobacterales*, etc.) are typical gut bacteria.

A distinctive feature of the microbiome of the Tom3 sample was a significant proportion of archaea, mainly methanogens. Probably, the high relative abundance of methanogens reflects local anaerobic conditions in the near-bottom sediment. This probably also explains the relatively high abundance of anaerobes of the phylum *Spirochaetota*, which was almost absent in the Tom2 sample.

Compared to the composition of the microbiomes revealed by 16S rRNA gene profiling, the resistomes represented the same main bacterial lineages, but the proportion of *Proteobacteria* was higher in the resistomes (Figure 1). Of the 726 resistance genes identified in the Tom2 sample, 121 genes were assigned to the family *Enterobacteriaceae* (mostly *Escherichia* and *Klebsiella*), whose share in the community, according to 16S RNA gene profiling data, was only 0.06%. Likewise, *Enterobacteriaceae* accounted for 26 ARG in the Tom3 sample (4% of the total), although this family accounted for only 0.03% of 16S rRNA gene sequences. *Enterobacteriaceae* are typical intestinal inhabitants and are considered as markers of fecal contamination [40]. Most of these genes code for an efflux pumps, but genes of enzymes that ensure the inactivation of aminoglycosides and beta-lactam antibiotics were also found.

One of detected strains carrying multiple resistance determinants (MAG-8), *Escherichia (Shigella) flexneri*, also represented *Enterobacteriaceae*. *Shigella* can cause moderate to acute gastrointestinal tract infection, known as shigellosis [41]. *Shigella* isolates could be resistant to various antibiotics, including sulfonamides, tetracycline, chloramphenicol, ampicillin, quinolones, and the third-generation cephalosporins [42,43,44,45], and several multidrug-resistant isolates have been characterized [41]. In particular, β-lactamases could provide high level resistance to penicillin and first-generation, second-generation and third-generation cephalosporins and monoamide antibiotics [46]. The presence of three class C beta-lactamases in MAG-8 along with numerous efflux pumps indicates that this bacterium is probably a multidrug-resistant strain that could disseminate ARGs in the manure storage lagoon.

The same is probably true for the second potential multidrug-resistant strain, although its taxonomic affiliation and origin is less clear. This strain, represented by MAG-42, was affiliated with the *Proteobacteria*, the family *Alcaligenaceae*. Although this family accounted for about 6.4% of the whole microbiome, the share of this strain was much lower (~0.3%), while MAGs representing dominant *Alcaligenaceae* phylotypes lacked recognizable ARGs. Multi-drug resistant pathogens from this family, e.g., *Alcaligenes faecalis*, have been repeatedly described [47,48,49,50]. The most common was resistance to beta-lactams, aminoglycosides, fluoroquinolone and tetracyclines. Particularly, ARGs were identified in *Alcaligenes* strains isolated form livestock manure [51], pigsties and manured soil [52]. Likewise, ARGs predicted to confer resistance to aminoglycosides, diaminopyrimidines, macrolide–lincosamide–streptogramin and tetracyclines were identified in MAG-42.

To assess the stability of the composition of microbial communities and their resistomes, we conducted a comparative analysis of manure samples taken from the same storage lagoon with an interval of two years. In both samples, three phyla, *Firmicutes*, *Proteobacteria* and *Bacteroidota*, accounted for more than half of the communities. A specific feature of the Tom2 sample is the presence of a significant fraction of methanogenic archaea, which is probably associated with the presence of anaerobic sediments in this sample. The composition of the resistome turned out to be more stable. Thus, genes from all 21 dominant AMR gene families (Figure 3) were found in both samples. The same can be said about the drug classes for which ARGs were found (Figure 2). The observed differences in the representation of different ARGs mainly reflected differences in the content of individual taxonomic groups. For example, the share of the RND antibiotic efflux pumps characteristic of *Proteobacteria* was higher in the resistome of the Tom2 sample, in which the relative abundance of *Poteobacteria* themselves was also higher.

## 5. Conclusions

16S rRNA gene profiling and metagenomic analysis revealed that both the prokaryotic community and the resistome were dominated by the *Firmicutes*, *Proteobacteria*, and *Bacteroidota*. However, the ARG relative abundance was higher in the *Proteobacteria* than in the *Firmicutes* and especially the *Bacteroidota*. Besides multidrug efflux pumps, the predominating ARGs potentially encoded resistance to tetracyclines, macrolide–lincosamide–streptogramin, aminoglycosides, peptide antibiotics, rifamycin, chloramphenicol, and beta-lactams. Genes from all predominant AMR gene families were found in samples collected with 2 years interval indicating overall stability of the resistome. Antibiotic efflux was the dominant mechanism of resistance in the *Proteobacteria*, while the *Firmicutes*, *Actinobacteriota* and *Bacteroidota* mostly rely on antibiotics inactivation, target alteration or protection. Metagenomics analysis indicated that the most numerous phylotypes lacked ARGs, while a limited number of lineages that presumably originated from the gut, but were relatively rare in the manure storage lagoon, were the main source of ARGs. Some of these strains are potential pathogens carrying multiple resistance determinants.

## Figures and Tables

**Table 1:** ARGs in multidrug-resistant strains in the Tom2 sample.

AMR Gene Family	Drug Class	Resistance Mechanism	Number of Genes in MAG-8	Number of Genes in MAG-42
ABC antibiotic efflux pump	various	efflux	3	0
MFS antibiotic efflux pump	various	efflux	12	1
RND antibiotic efflux pump	various	efflux	22	0
General bacterial porin with reduced permeability to beta-lactams	beta-lactams	reduced permeability	3	0
ampC-type beta-lactamase	cephalosporin; penam	inactivation	1	0
EC beta-lactamase	cephalosporin	inactivation	2	0
Pmrphosphoethanolamine transferase	peptide antibiotics	target alteration	2	0
Undecaprenyl pyrophosphate related proteins	peptide antibiotics	target alteration	1	0
ANT(6)	aminoglycosides	inactivation	0	1
ANT(9)	aminoglycosides	inactivation	0	1
APH(2″)	aminoglycosides	inactivation	0	1
APH(3″)	aminoglycosides	inactivation	0	1
Trimethoprim resistant dihydrofolate reductase	diaminopyrimidine	target replacement	0	2
Lincosamidenucleotidyltransferase	lincosamide	inactivation	0	1
Non-erm 23S ribosomal RNA methyltransferase	macrolide- lincosamide-streptogramin	target alteration	0	2
Erm 23S ribosomal RNA methyltransferase	macrolide- lincosamide-streptogramin	target alteration	0	5
Tetracycline-resistant ribosomal protection protein	tetracycline	target protection	0	3
**Total**			**46**	**18**

Abbreviations: ARG, antibiotic resistance genes; AMR, antimicrobial resistance; ABC, ATP-binding cassette; MFS, major facilitator superfamily; RND, resistance-nodulation-division; MAG, metagenome-assembled genome.

## Data Availability

The raw sequencing data have been deposited in the NCBI Sequence Read Archive (SRA) under the accession numbers SRR21185896 (16S rRNA gene fragments, Tom2 sample), SRR21203432 (metagenome, Tom2 sample), SRR13442339 (16S rRNA gene fragments, Tom3 sample), and SRR13442943 (metagenome, Tom3 sample) within the BioProject PRJNA785864.

## Supplementary Materials

The following supporting information can be downloaded at: https://www.mdpi.com/article/10.3390/microorganisms10112301/s1, Table S1: ARGs identified in the metagenome of manure the Tom2 sample; Table S2: ARGs identified in the metagenome of manure the Tom3 sample.
